# Innovations in heart failure management: The role of cutting-edge biomarkers and multi-omics integration^☆^

**DOI:** 10.1016/j.jmccpl.2025.100290

**Published:** 2025-03-01

**Authors:** Jose Mesquita Bastos, Beatriz Colaço, Rui Baptista, Cristina Gavina, Rui Vitorino

**Affiliations:** a Department of Medical Sciences, Institute of Biomedicine iBiMED, University of Aveiro, 3810-193 Aveiro, Portugal; b Cardiovascular R&D Centre - UnIC@RISE, Department of Surgery and Physiology, Faculty of Medicine of the University of Porto, 4200-319 Porto, Portugal; c Cardiology Department, Hospital Infante D. Pedro, Centro Hospitalar do Baixo Vouga, Aveiro, Portugal; d Department of Cardiology, Centro Hospitalar de Entre o Douro e Vouga, Santa Maria da Feira, Portugal; e Faculty of Medicine, University of Coimbra, Coimbra, Portugal; f University of Coimbra, Center for Innovative Biomedicine and Biotechnology (CIBB), Coimbra, Portugal; g Clinical Academic Center of Coimbra (CACC), Coimbra, Portugal; h Pedro Hispano Hospital - ULS Matosinhos, Matosinhos, Portugal; i Cardiology Department, Faculty of Medicine, University of Porto, Oporto, Portugal; j RISE- Health Research Network, Faculty of Medicine, University of Porto, Oporto, Portugal

**Keywords:** Multi-omics, Biomarkers, Proteomics, Lipidomics, Metabolomics, Data complexity, Advanced bioinformatics, Personalized medicine, Diagnostic tools

## Abstract

Heart failure (HF) remains a major cause of morbidity and mortality worldwide and represents a major challenge for diagnosis, prognosis and treatment due to its heterogeneity. Traditional biomarkers such as BNP and NT-proBNP are valuable but insufficient to capture the complexity of HF, especially phenotypes such as HF with preserved ejection fraction (HFpEF). Recent advances in multi-omics technology and novel biomarkers such as cell-free DNA (cfDNA), microRNAs (miRNAs), ST2 and galectin-3 offer transformative potential for HF management. This review explores the integration of these innovative biomarkers into clinical practice and highlights their benefits, such as improved diagnostic accuracy, enhanced risk stratification and non-invasive monitoring capabilities. By leveraging multi-omics approaches, including lipidomics and metabolomics, clinicians can uncover new pathways, refine the classification of HF phenotypes, and develop personalized therapeutic strategies tailored to individual patient profiles. Remarkable advances in proteomics and metabolomics have identified biomarkers associated with key HF mechanisms such as mitochondrial dysfunction, inflammation and fibrosis, paving the way for targeted therapies and early interventions. Despite the promising results, significant challenges remain in translating these findings into routine care, including high costs, technical limitations and the need for large-scale validation studies. This report argues for an integrative, multi-omics-based model to overcome these obstacles and emphasizes the importance of collaboration between researchers, clinicians and policy makers. By linking innovative science with practical applications, multi-omics approaches have the potential to redefine HF management and lead to better patient outcomes and more sustainable healthcare systems.

## Highlights

• Emerging biomarkers: The combination of BNP/NT-proBNP with novel markers (galectin-3, ST2, cfDNA) improves HF diagnosis and prognosis.
• Multi-omics reveals HF mechanisms (oxidative stress, inflammation, overload) for targeted biomarker.
• Biomarkers help in HF diagnosis, risk stratification and therapy monitoring for early, personalized treatment.
• The integration of multi-omics faces technical, financial and ethical challenges that limit its widespread use in HF management.
• Future HF research will focus on specific workflows, scalable technologies and collaborations to translate results into clinical practice.

## 1 Introduction

Heart failure (HF) is a major global health problem affecting over 64 million people worldwide [1]. It remains a major cause of morbidity, mortality and healthcare costs. Its prevalence is expected to increase due to factors such as the ageing population, improved survival rates from other cardiovascular diseases and the increasing burden of risk factors such as obesity, diabetes and hypertension [2,3]. HF is a complex clinical syndrome characterized by structural or functional impairment of ventricular filling or ejection. It is usually classified into three categories based on left ventricular ejection fraction (LVEF): reduced ejection fraction (HFrEF, LVEF <40 %), mildly reduced ejection fraction (HFmEF, LVEF 41–49 %) and preserved ejection fraction (HFpEF, LVEF ≥50 %). HFpEF poses a special challenge in diagnosis and treatment due to its heterogeneity and the overlap of clinical features with other diseases. The prevention and early identification of HF remain critical priorities in clinical care. Primary prevention focuses on mitigating modifiable risk factors such as hypertension, diabetes, and obesity, while early risk stratification seeks to identify individuals at high risk of developing HF. Current models for HF risk prediction integrate clinical, imaging, and biomarker data. Traditional tools such as the Framingham Heart Study risk score provide general cardiovascular risk assessment but lack specificity for HF [4,5].

Despite advances in therapeutic strategies, HF continues to have a poor prognosis, with a five-year survival rate comparable to that of many cancers [6]. Current diagnostic and prognostic tools such as clinical assessments, imaging and biomarkers such as B-type natriuretic peptide (BNP) and N-terminal proBNP (NT-proBNP) have their limitations [7]. While valuable, these traditional biomarkers often fail to capture the systemic and molecular complexity of HF, particularly in HFpEF, where their diagnostic accuracy is diminished due to variability in patient populations with comorbidities such as obesity and chronic kidney disease [[8], [9], [10], [11]]. This highlights the urgent need for innovative approaches to better stratify risk, diagnose HF phenotypes and guide personalized treatments.

Emerging multi-omics technologies, including genomics, transcriptomics, proteomics, metabolomics and lipidomics, offer transformative potential to address these gaps. By integrating data from these platforms, multi-omics approaches provide a comprehensive understanding of the pathophysiology of HF and enable the discovery of novel biomarkers and therapeutic targets [12,13]. This review aims to highlight the transformative potential of multi-omics and novel biomarkers in addressing the diagnostic, prognostic and therapeutic challenges of HF. By exploring emerging trends and technologies, we emphasize the importance of linking advanced biomarker research with clinical practice to improve patient outcomes and usher in a new era of precision cardiovascular medicine.

## 2 Traditional biomarkers in HF

### 2.1 Role and limitations of natriuretic peptides

Natriuretic peptides, including B-type natriuretic peptide (BNP) and its inactive precursor NT-proBNP, are important biomarkers for the diagnosis and treatment of heart failure (HF). These peptides, which are secreted by cardiomyocytes in response to volume or pressure overload-induced stress on the myocardial wall, provide clinicians with robust diagnostic tools to differentiate HF from other causes of dyspnea in both acute and chronic stages. In addition, BNP and NT-proBNP are invaluable for monitoring disease progression, adjusting treatment and predicting adverse outcomes such as hospitalization, readmission and mortality [14].

In clinical practice, natriuretic peptides are integrated into HF guidelines and decision-making frameworks. Elevated BNP or NT-proBNP levels confirm the diagnosis of HF in symptomatic patients, assess disease severity and help with risk stratification. Conversely, low values are particularly useful for ruling out HF due to their high negative predictive value [15]. This dual benefit makes BNP and NT-proBNP indispensable, especially in the emergency department and outpatient setting where timely and accurate diagnosis is critical. However, despite their established role, natriuretic peptides also have significant limitations. One notable challenge is their lack of specificity for HF. BNP and NT-proBNP levels may increase due to non-HF-related conditions such as renal dysfunction, pulmonary hypertension, sepsis and advanced age [16,17]. This lack of specificity can complicate the diagnostic process, especially in patients with multiple comorbidities. In addition, levels may be falsely low in obese individuals [18], which can lead to underdiagnosis and delayed treatment.

The diagnostic utility of BNP and NT-proBNP also varies depending on the phenotype of HF. In heart failure with preserved ejection fraction (HFpEF), which accounts for almost half of all HF cases, these biomarkers are usually not as elevated as in heart failure with reduced ejection fraction (HFrEF) [19]. HFpEF is characterized by an almost normal ejection fraction and significant diastolic dysfunction. The relatively low myocardial wall stress results in BNP and NT-proBNP values that may not reach diagnostic thresholds, except in the presence of congestion. This challenge is compounded in obese patients, in whom biomarker concentrations are often suppressed. Consequently, these limitations may lead to diagnostic inaccuracies and suboptimal care for HFpEF patients [20]. The inclusion of BNP and NT-proBNP serves to provide a critical baseline reference for new proteomic biomarkers. By highlighting their strengths and limitations, we aim to emphasize the need for new biomarkers that address these gaps, particularly in challenging contexts such as HFpEF, obesity and multimorbid patient populations.

Atrial natriuretic peptide (ANP) and adrenomedullin (ADM) are additional biomarkers of clinical importance for the treatment of heart failure (HF). ANP is primarily secreted by atrial myocytes in response to volume overload and increased atrial pressure. Its vasodilatory and natriuretic effects help to reduce cardiac preload and afterload. ANP levels provide valuable information on hemodynamic stress and correlate with the severity of HF. However, its shorter half-life and rapid degradation in the circulation have limited its clinical utility as a stand-alone biomarker. ADM, on the other hand, is a potent vasodilator peptide produced by vascular endothelial and smooth muscle cells [15,21]. It plays an important role in reducing systemic vascular resistance and increasing cardiac output. Elevated ADM levels are strongly associated with the progression of HF and adverse outcomes. In contrast to BNP and NT-proBNP, ADM is influenced by oxidative stress and inflammation, providing complementary insights into the pathophysiology of HF [15]. Both biomarkers have been shown to have prognostic value and can be used to guide therapy in HF patients. Research suggests that integrating ADM and ANP measurements with traditional markers such as BNP and NT-proBNP could improve diagnostic accuracy and risk stratification [22]. The use of these markers in clinical practice is promising, but further validation in large-scale studies is needed to standardize their use.

### 2.2 Challenges in the diagnosis and risk stratification of HFpEF

The diagnosis of HFpEF is challenging due to its complex pathophysiology and the overlap of clinical features with other diseases such as chronic obstructive pulmonary disease (COPD), anemia and obesity. In contrast to HFrEF, where natriuretic peptides reliably reflect left ventricular dysfunction, HFpEF encompasses a spectrum of abnormalities, including systemic inflammation, endothelial dysfunction and metabolic disturbances, which are less directly associated with BNP and NT-proBNP levels [5,23]. Consequently, looking at natriuretic peptides alone often leads to diagnostic uncertainty, especially in the presence of confounding factors.

In clinical practice, the diagnostic process for HFpEF usually involves a combination of clinical assessment, imaging (e.g. echocardiography to identify increase left atrial volumes and left ventricular mass, as well as signs of diastolic dysfunction) and natriuretic peptide evaluation as a surrogate of congestion. However, the standard diagnostic algorithms often fail in borderline cases where natriuretic peptide levels are only slightly elevated or within the normal range. For example, obese patients with HFpEF may have BNP levels below the diagnostic threshold even when significant cardiac dysfunction is present [24,25]. Conversely, patients with chronic kidney disease may have elevated BNP levels due to impaired renal clearance despite the absence of significant cardiac abnormalities. These inconsistencies highlight the limitations of BNP and NT-proBNP as stand-alone tools for the diagnosis of HFpEF [26,27]. In addition to diagnosis, risk stratification in HFpEF is associated with additional difficulties. Although elevated BNP and NT-proBNP levels are associated with worse outcomes, including hospitalization and mortality, they do not capture the broader spectrum of pathophysiological processes underlying HFpEF. Unlike HFrEF, where these biomarkers reliably predict outcomes, HFpEF involves multiple mechanisms such as microvascular dysfunction, systemic inflammation and myocardial fibrosis that are poorly captured by natriuretic peptide concentrations. This limits their utility in guiding treatment decisions and predicting long-term outcomes in HFpEF patients [28,29].

## 3 Emerging biomarkers in HF

### 3.1 Genomic and transcriptomic markers

Genomic and transcriptomic markers have significantly advanced HF research and treatment by providing unprecedented insights into the molecular complexity of the disease. These markers play a critical role in understanding the heterogeneity of HF, refining disease classification, and preparing for precision medicine. By identifying key genetic variants and transcriptomic regulators, researchers can uncover new pathways and biomarkers that contribute to disease progression, therapeutic response, and risk stratification.

One of the most studied genomic biomarkers in HF is single nucleotide polymorphisms (SNPs), which represent genetic variations that affect individual susceptibility, disease progression, and response to treatment. SNPs have provided valuable insights into subtype-specific mechanisms. In HFpEF, genetic variations in genes associated with inflammation and fibrosis, such as the interleukin-6 receptor (IL6R) gene, elucidate the systemic inflammatory and fibrosis mechanisms underlying the pathophysiology. In HFrEF, polymorphisms in the beta-adrenergic receptor gene (ADRB1), particularly the Arg389Gly variant, affect therapeutic response to beta-blockers. The Arg389 variant is associated with enhanced beta-adrenergic signaling, leading to increased myocardial contractility and greater sensitivity to beta-blockers. As a result, HFrEF patients carrying the Arg389 variant often have improved left ventricular ejection fraction and a lower risk of death compared to Gly389 carriers [30]. In contrast, the Gly389 variant is associated with reduced receptor activity and weaker response to beta-blocker therapy, highlighting the importance of genotype-guided treatment strategies [31]. In addition, genetic variations in the angiotensin-converting enzyme (ACE) gene, such as the insertion/deletion (I/D) polymorphism, have significant effects on the renin-angiotensin-aldosterone system (RAAS). The D allele is associated with increased circulating ACE levels, leading to higher angiotensin II production, which contributes to myocardial remodeling and worsening heart failure outcomes. Consequently, HFrEF patients with the DD genotype may derive greater benefit from ACE inhibitors, highlighting the potential of personalized RAAS-targeted therapy in the treatment of HF [32].

In addition to SNPs, cell-free DNA (cfDNA) has emerged as a promising non-invasive biomarker in HF. cfDNA is released during cellular apoptosis or necrosis, with its concentration correlating with the degree of tissue damage and systemic inflammation. In HFrEF, elevated cfDNA levels provide insight into myocardial damage and disease severity, while in HFpEF, cfDNA is associated with endothelial dysfunction and microvascular pathology. Integrating cfDNA with transcriptomic profiling improves risk stratification and prognostic accuracy in all HF subtypes, especially in complex or borderline cases. Genome-wide association studies (GWAS) have also identified genetic loci associated with myocardial stress and fibrosis, such as TTN and BAG3, which further complement transcriptomic biomarkers for improved risk prediction and therapeutic targeting [33].

Besides genomic variants, transcriptomic markers, particularly non-coding RNAs (ncRNAs), have gained attention due to their regulatory role in HF pathophysiology. Non-coding RNAs, including microRNAs (miRNAs), long non-coding RNAs (lncRNAs), and circular RNAs (circRNAs), are important post-transcriptional regulators that modulate gene expression, cardiac remodeling, and inflammation in HF. Among them, microRNAs (miRNAs) are well-established biomarkers due to their stability in the circulation and tissue-specific expression. Certain miRNAs show subtype-specific significance. In HFpEF, miR-223 and miR-29 are strongly associated with myocardial fibrosis and systemic inflammation, two hallmarks of this HF subtype [34,35]. In addition, miR-503-5p and miR-193a-5p have been identified as promising biomarkers for distinguishing HFpEF from HFrEF [36,37]. In HFrEF, miR-208 and miR-499 are associated with cardiac remodeling, myocardial stress and apoptosis, making them valuable indicators of disease severity and progression [38]. The integration of miRNA panels with conventional biomarkers such as BNP and NT-proBNP significantly increases diagnostic precision and improves risk stratification in HF, especially in cases with unclear clinical presentation or overlapping symptoms [39].

In addition to miRNAs, long non-coding RNAs (lncRNAs) and circular RNAs (circRNAs) are gaining interest as new transcriptomic markers in HF. Although few lncRNAs and circRNAs have been thoroughly characterized in the context of HF, their role in regulating fibrosis, hypertrophy and metabolism suggests clinical potential [40]. Some lncRNAs, such as LIPCAR (long intergenic non-coding RNA predicting cardiac remodeling), have been associated with unfavorable ventricular remodeling and poor prognosis in HF patients. CircRNAs, which act as sponges for miRNAs, influence gene expression and cellular signaling. In HF, circRNAs such as circNFIB and circSLC8A1 have been associated with myocardial fibrosis and apoptosis, although further studies are needed to determine their diagnostic and prognostic utility [41]. By incorporating miRNAs, lncRNAs and circRNAs into multi-omics platforms, researchers can bridge the gap between genomic predisposition and transcriptomic alterations, leading to refined stratification of HF subtypes and improved biomarker-based clinical decision making.

Despite the remarkable potential of genomic and transcriptomic markers in HF, several challenges remain. The integration of these markers into clinical practice is hampered by factors such as assay standardization, high costs and the need for large-scale validation in diverse populations. In addition, advanced bioinformatics tools are required to analyze and interpret high-dimensional data and ensure that the findings from genomic and transcriptomic studies can be translated into actionable clinical strategies. Future research should focus on expanding the catalog of lncRNAs and circRNAs associated with HF, validating multi-omics panels that integrate genetic variants, cfDNA and ncRNAs for personalized risk assessment, and developing cost-effective high-throughput sequencing methods to facilitate clinical translation. By addressing these challenges, genomic and transcriptomic markers, especially in conjunction with multi-omics, will play a critical role in the next generation of HF diagnostics, risk stratification and targeted therapies.

### 3.2 Proteomic markers

Proteomics, the study of protein profiles and functions, has proven to be an important tool for understanding HF and its different subtypes, HFpEF and HFrEF. By analyzing proteins in body fluids and tissues, proteomics provides deep insights into the molecular mechanisms underlying HF and enables the identification of biomarkers that inform diagnosis, risk stratification and therapeutic strategies. This discipline comprises two main strategies: discovery proteomics and targeted proteomics, each of which offers unique insights into protein function and disease mechanisms.

**Fig. 1:**
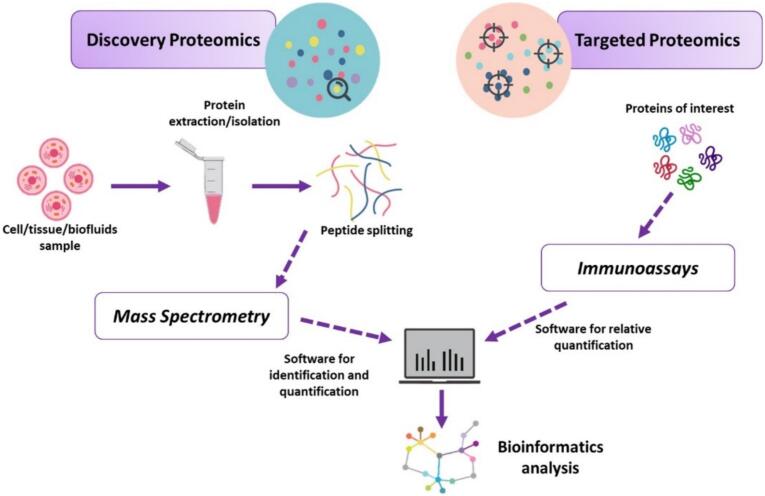
Schematic representation of the proteomic analysis approaches used in heart failure research. The figure illustrates the two main proteomics methods: discovery proteomics, which uses high-throughput techniques such as mass spectrometry (MS) to identify and characterize a large number of proteins without prior selection, and targeted proteomics, which focuses on the precise quantification of specific proteins using techniques such as selective reaction monitoring (SRM) and parallel reaction monitoring (PRM). These approaches contribute to the identification of novel biomarkers and enable improved diagnosis, risk stratification and therapy monitoring in patients with heart failure.

Discovery proteomics takes an untargeted approach that aims to identify and characterize a large number of proteins simultaneously. This process includes sample preparation, protein separation, identification and validation. Techniques such as gel electrophoresis and high-resolution liquid chromatography (HPLC) are used to separate proteins based on their physicochemical properties such as size, charge and affinity. Mass spectrometry (MS) is then used to determine the mass-to-charge ratio (*m/z*) of the peptides, enabling the identification and characterization of the proteins [42,43]. Approaches such as top-down proteomics analyze intact proteins and provide insights into post-translational modifications, while bottom-up proteomics involves enzymatic digestion of proteins into peptides for analysis and provides detailed sequence information. These methods, complemented by advanced techniques such as tandem MS (MS/MS) and data-independent acquisition (DIA), have facilitated the identification of numerous RF-related biomarkers [[44], [45], [46]]. Targeted proteomics focuses on the precise quantification and analysis of specific proteins of interest, bridging the gap between discovery and clinical application. Techniques such as selective reaction monitoring (SRM) and parallel reaction monitoring (PRM) offer high sensitivity and reproducibility for the detection of biomarker candidates identified in discovery studies. The validation of these biomarkers is of critical importance in order to facilitate the translation of proteomic discoveries into tools for the management of heart failure [47,48] (Fig. 1).

Among the most important proteomic biomarkers, galectin-3 (Gal-3) has shown for HF management. Gal-3 is closely associated with inflammation and fibrosis, processes that are particularly pronounced in HFpEF. Elevated Gal-3 levels correlate with myocardial stiffness and impaired relaxation, the characteristic features of HFpEF. In HFrEF, Gal-3 is associated with unfavorable remodeling and progression of HF. Its dual role as a diagnostic and prognostic biomarker and as a therapeutic target for antifibrotic strategies underscores its importance in all HF phenotypes [49,50]. Nevertheless, they retain their diagnostic utility, especially when combined with other markers such as Gal-3 or high-sensitivity C-reactive protein (hs-CRP) to rule out confounding factors such as obesity or renal dysfunction [51].

Soluble ST2 (sST2) is another proteomic biomarker with pronounced utility in HF. As a marker of myocardial stress and fibrosis, ST2 is particularly valuable in HFpEF as it reflects underlying fibrotic and inflammatory mechanisms that are less directly associated with natriuretic peptide levels [52]. In HFrEF, elevated ST2 levels correlate with poor outcomes, including increased mortality and hospitalization. In contrast to natriuretic peptides, ST2 is not significantly affected by factors such as renal insufficiency or obesity, making it a reliable biomarker for different patient groups [53,54].

The role of TIMP-1 (Tissue Inhibitor of Metalloproteinases-1) in HF underscores the importance of proteomic biomarkers for understanding extracellular matrix (ECM) remodeling. TIMP-1 regulates ECM dynamics, a crucial process in HF [55]. Elevated TIMP-1 levels in HFpEF reflect pathologic fibrosis that contributes to diastolic dysfunction and myocardial stiffening. In HFrEF, TIMP-1 levels are associated with unfavorable ventricular remodeling and impaired cardiac function, highlighting its potential as a prognostic biomarker and therapeutic target for fibrosis modulation [56].

Other notable biomarkers include cystatin C (CysC) [57], a marker of renal function [58], and high-sensitivity C-reactive protein (hs-CRP), a marker of systemic inflammation [59]. CysC is particularly important in HFpEF, where renal dysfunction is a common comorbidity and signals worsening outcomes. In HFrEF, CysC provides insight into the interplay between kidney and heart health, particularly in patients with cardio-renal syndromes [60].

### 3.3 Lipidomics markers

Lipidomics, the comprehensive study of lipid profiles and their functional role, has become a central area for understanding HF and its phenotypes. Lipids play a critical role in cellular structures, energy storage and signaling processes, and their dysregulation is a hallmark of HF progression. Advanced lipid profiling has identified several lipid biomarkers that are relevant to different HF phenotypes and provide valuable insights into the metabolic shifts underlying the disease [61].

Among the most critical lipid biomarkers, cardiolipins stand out due to their essential role in mitochondrial function and energy production. These unique phospholipids, which are mainly localized in the inner mitochondrial membrane, stabilize protein complexes involved in oxidative phosphorylation, the major energy-generating process in cardiomyocytes. In HF, especially HFrEF, cardiolipin integrity is often compromised, leading to mitochondrial dysfunction and energy deficit that exacerbates impaired cardiac contractility [62,63]. In addition, cardiolipin abnormalities trigger cardiomyocyte apoptosis, contributing to unfavorable cardiac remodeling. In HFpEF, where energy metabolism is less directly affected but still critical, cardiolipin levels may reflect subclinical mitochondrial dysfunction and serve as a biomarker for early detection and possible therapeutic intervention to restore energy balance [64,65].

Sphingolipids, another important class of lipids, are bioactive molecules involved in inflammation, apoptosis and cellular homeostasis. In HF, disturbances in sphingolipid metabolism, including elevated ceramide levels and altered sphingosine-1-phosphate pathways, contribute to inflammatory signaling and apoptotic cell death [66]. In HFpEF, these perturbations exacerbate endothelial dysfunction and systemic inflammation, the main hallmarks of this subtype. In HFrEF, the inflammatory cascade triggered by sphingolipids accelerates myocardial damage and fibrosis, highlighting its utility as a biomarker for disease progression and as a target for therapeutic strategies to curb inflammation and fibrosis [67].

Lysophosphatidylcholine (LPC) 18:2, a glycerophospholipid, has also been shown to be an important biomarker in HF. Elevated levels of LPC 18:2 are closely associated with systemic inflammation and impaired lipid metabolism. Mechanistically, LPC 18:2 promotes endothelial dysfunction and oxidative stress, which exacerbates vascular and myocardial damage. In HFpEF, where the vascular contribution to pathophysiology is pronounced, LPC 18:2 levels may correlate with microvascular dysfunction and disease severity. In HFrEF, its increase reflects increased oxidative stress and inflammatory responses, making it a valuable marker for monitoring disease progression and therapeutic efficacy [68].

The balance between omega-3 and omega-6 fatty acids also plays a critical role in cardiovascular health and the progression of HF [69]. Omega-3 fatty acids such as eicosapentaenoic acid (EPA) and docosahexaenoic acid (DHA) have anti-inflammatory and cardioprotective effects, while an excess of omega-6 fatty acids such as arachidonic acid promotes pro-inflammatory responses. In HF, this balance is often disturbed, with a relative deficiency of omega-3 fatty acids and an excess of omega-6 fatty acids, leading to chronic inflammation and oxidative stress [70]. In HFpEF, where systemic inflammation is an important pathophysiological factor, restoring this balance through omega-3 supplementation has shown the potential to improve endothelial function and reduce disease progression. In HFrEF, these fatty acids may attenuate myocardial stress and inflammatory damage, providing a complementary approach to conventional therapies.

Lipidomics provides a unique window into mitochondrial dysfunction, inflammation and systemic metabolic dysfunction in HF. Advances in techniques such as high-resolution mass spectrometry have made it possible to integrate lipidomic data into clinical practice, improving diagnostic precision and risk stratification [71]. Future research should focus on validating these lipid biomarkers in different HF populations and integrating them into multi-omics approaches to develop personalized therapeutic strategies. By utilizing lipidomic insights, clinicians can better address the heterogeneity of HF phenotypes and improve outcomes for patients with this complex disease.

### 3.4 Metabolomics markers

Metabolomics, the study of small molecule metabolites in biological systems, plays a critical role in understanding the metabolic disorders associated with HF. These disorders often reflect broader pathological processes, including mitochondrial dysfunction, impaired energy metabolism and abnormal substrate utilization. Metabolomics markers provide a detailed biochemical snapshot that offers insight into the severity and progression of HF while serving as potential therapeutic targets. Among the most studied markers are amino acids, acylcarnitines and butyrylcarnitine, each of which reveal specific aspects of metabolic dysregulation in HF [72].

Amino acids, particularly branched-chain amino acids (BCAAs) such as leucine, isoleucine and valine, are vital for energy metabolism, protein synthesis and the maintenance of cellular homeostasis. In the context of HF, altered BCAA levels have been identified as key indicators of metabolic dysfunction. Elevated BCAA levels have been observed in HF patients, possibly due to impaired oxidative metabolic pathways and increased myocardial dependence on alternative energy sources. These changes correlate with the severity of HF as the heart struggles to maintain its energy balance under stress [73].

In addition to BCAAs, changes in other amino acids, such as glutamate and alanine, underscore the metabolic stress experienced by HF patients. These metabolites reflect shifts in nitrogen metabolism and gluconeogenesis, processes that are often upregulated as compensatory mechanisms in the failing heart. By assessing amino acid profiles, clinicians can gain a deeper understanding of the metabolic state of HF patients, allowing stratification based on disease severity and potential metabolic vulnerabilities [74].

Acylcarnitines serve as important intermediates in fatty acid oxidation and mitochondrial energy production. In HF, elevated levels of short- and medium-chain acylcarnitines are frequently detected, indicating mitochondrial inefficiencies and incomplete fatty acid oxidation. This mitochondrial dysfunction is a hallmark of HF pathology, contributing to impaired ATP production and exacerbating cardiac stress. Elevated acylcarnitine levels are also associated with systemic metabolic disorders such as insulin resistance, further complicating the treatment of HF [75]. The accumulation of acylcarnitines is not only a diagnostic marker but also has prognostic significance. Higher acylcarnitine levels have been associated with poorer clinical outcomes, including higher hospitalization rates and mortality in HF patients. These findings emphasize the potential of acylcarnitines as biomarkers for risk stratification and as targets for therapies aimed at improving mitochondrial function [76].

Butyrylcarnitine, a specific subtype of acylcarnitine, provides information about abnormalities in fatty acid metabolism. In HF, butyrylcarnitine levels are often disturbed, which is due to disturbances in the beta-oxidation pathway and energy production. The failing heart, faced with an energy deficit, shifts its dependence to less efficient metabolic pathways, leading to an accumulation of intermediates such as butyrylcarnitine [77]. This biomarker is associated with reduced myocardial efficiency and impaired cardiac function, making it a valuable indicator of metabolic problems in HF patients. Its levels also correlate with disease severity, suggesting its usefulness in grading HF and monitoring response to therapy. The analysis of metabolomics markers, including amino acids, acylcarnitines and butyrylcarnitine, offers significant clinical benefits in the management of HF. One of the most important benefits is improved diagnostic accuracy. By providing a detailed assessment of metabolic health, metabolomics enables the detection of disorders that often occur before clinical symptoms [78]. This is particularly important in HF patients with HFpEF, a subgroup where conventional biomarkers often lack the sensitivity or specificity required for early and accurate diagnosis. Metabolomics bridges this gap, improving diagnostic accuracy and facilitating timely intervention.

The prognostic utility of metabolomics markers further enhances their clinical relevance. The strong correlation between these markers and the severity or outcome of HF allows for better risk stratification. For example, patients with elevated acylcarnitines or significantly altered amino acid profiles can be identified as having a higher risk of adverse events such as hospitalization or disease progression [79]. Such findings help physicians apply more aggressive monitoring or intervention strategies for these high-risk patients, ultimately improving their clinical outcomes.

### 3.5 Current limitations

**Fig. 2:**
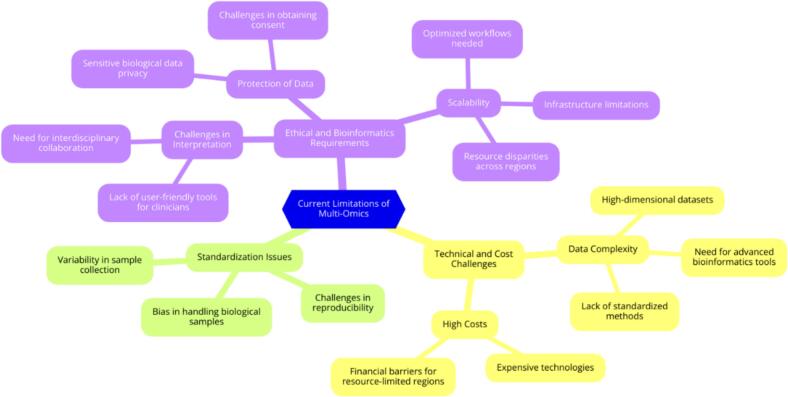
Illustrates the various challenges associated with the application of multi-omics in the HF clinical and research environment. The color coding in the figure represents different categories of challenges: Purple highlights ethical and bioinformatics challenges, such as privacy, consent, and the need for interdisciplinary collaboration to interpret complex datasets; Green represents standardization issues, including variability in sample collection, bias in handling biological samples, and challenges in ensuring reproducibility; Yellow focuses on technical and financial challenges, including high costs, expensive technologies, and the complexity of data that require advanced bioinformatics tools and standardized methods. These categories emphasize the importance of overcoming these challenges to enable effective and scalable implementation of multi-omics.

Although the potential of multi-omics to transform the treatment of HF is considerable, its widespread application faces significant challenges. Addressing these issues is critical for effective translation into clinical practice, particularly for stratification of HF phenotypes such as HFrEF and HFpEF [80]. Multi-omics integrates different data sets, including genomics, proteomics, metabolomics, lipidomics and transcriptomics, providing a comprehensive view of the pathophysiology of HF [81] (Fig. 2). Genome-wide association studies (GWAS) complement multimicroscopy by identifying genetic variants associated with HF risk and progression. For example, GWAS analyzes have uncovered loci associated with myocardial stress, fibrosis and inflammation. Genetic risk markers such as SNPs have been identified in genes such as TTN, BAG3 and NPPA [82]. These findings enrich the understanding of the relationship between genotypeand phenotype and improve the specificity of biomarkers and the differentiation of HF subtypes.

Current multi-omics techniques include next-generation sequencing (NGS) for genomics, mass spectrometry for proteomics and metabolomics, and nuclear magnetic resonance (NMR) spectroscopy for lipidomics [83]. These approaches have led to the discovery of promising biomarkers, such as cell-free DNA (cfDNA), specific microRNAs (e.g. miR-223, miR-29), soluble ST2 and Gal-3. However, integrating this high-dimensional data into actionable insights for RF management remains a formidable challenge. The complexity of multi-omics data is compounded by the heterogeneity of HF pathophysiology and requires specialized bioinformatics tools and computational infrastructure. In addition, gaps in the standardization of data integration methods hinder efforts to achieve reproducible and clinically relevant results.

The implementation of multi-omics in RF treatment is also limited by the significant costs associated with advanced technologies such as NGS and high-resolution mass spectrometry. These financial barriers limit access to multi-omics-based diagnostics, particularly in resource-poor healthcare systems where the burden of HF is disproportionately high. The development of cost-effective pipelines and scalable workflows is essential to expand access.

Standardization of the entire multi-omics pipeline is critical to enable reliable stratification of HF subtypes. Inconsistencies in sample collection methods — such as the timing or handling of blood samples — can significantly affect the reliability of metabolomic and proteomic data used to identify biomarkers [84]. In HFrEF and HFpEF, this variability affects the reproducibility of subtype-specific markers and limits the generalizability of results. The introduction of standardized protocols in all phases, from sample collection to data interpretation, is essential for clinical implementation.

Multi-omics research also involves large amounts of sensitive patient data, which raises significant ethical and legal concerns [85]. Robust safeguards are required to ensure compliance with data protection standards and prevent breaches. In addition, transforming complex datasets into clinically actionable insights remains a bottleneck, especially in distinguishing molecular signatures for HFrEF and HFpEF. User-friendly bioinformatics tools tailored to clinicians are urgently needed to facilitate the integration of multi-omics data into HF care [86].

Despite these challenges, the integration of multi-omics and GWAS into HF management offers transformative opportunities. GWAS results can be combined with proteomic and metabolomic biomarkers to improve personalized diagnostics and refine stratification of HF patients based on genetic predispositions. Dynamic risk prediction models utilizing GWAS-derived genetic variants and biomarkers such as ST2 and cfDNA can support therapeutic decision making. In addition, clinical decision support systems (CDSS) that incorporate multi-omics and GWAS data enable real-time risk stratification and personalized treatment planning, especially for HFpEF patients. By addressing these challenges, the integration of multi-omics with GWAS holds significant promise for the advancement of precision medicine in HF management. This approach paves the way for more targeted diagnostics, personalized treatment strategies and improved patient outcomes, which will ultimately transform HF care.

## 4 Clinical implementation of biomarkers and multi-omics

The clinical integration of novel biomarkers such as cfDNA, miRNAs, ST2 and Gal-3 in combination with multi-omics approaches represents a fundamental change in the treatment of HF. However, to achieve this, a structured framework is needed to effectively incorporate these advances into routine clinical care.

The first step in this process is to conduct comprehensive validation studies to determine the clinical utility of these biomarkers. Validation should focus on their sensitivity, specificity and reproducibility in different patient populations and HF subtypes. For example, cfDNA assays have shown strong correlations with cardiac injury and systemic inflammation, but their integration into standard protocols requires standardized platforms and harmonized assay techniques. Similarly, miRNA panels, which have been shown to be extremely useful in distinguishing HF phenotypes such as HFpEF and HFrEF, require rigorous evaluation in multicenter studies to ensure their robustness in practice.

Standardization also extends to laboratory workflows, where protocols need to be developed to facilitate the seamless adoption of these biomarkers. For example, cfDNA quantification could benefit from the integration of advanced sequencing methods, while miRNA-based diagnostics could utilize high-throughput qPCR platforms for rapid and cost-effective implementation. Scalability of such systems will be key to ensure accessibility and consistency across different clinical settings.

The use of electronic health records (EHRs) offers another layer of integration that allows these biomarkers to be incorporated into patient care pathways. Advanced data analytics and machine learning algorithms can analyze biomarker trends over time and provide actionable insights to clinicians. For example, a significant increase in Gal-3 levels in a patient who is routinely monitored could trigger an alert in the EHR system, prompting physicians to reassess inflammatory or fibrotic processes and adjust therapeutic strategies accordingly. This real-time integration not only improves patient outcomes, but also increases the efficiency of clinical decision-making. The path to integrating multi-omics into clinical practice requires a multidisciplinary approach. Collaboration between cardiologists, molecular biologists, bioinformaticians and healthcare administrators is critical to interpreting the complex data sets generated by multi-omics platforms. By synthesizing genomic, proteomic, lipidomic and metabolomic data, these teams can generate actionable insights that lead to personalized treatment strategies. For example, by integrating metabolome and lipidome profiles with genomic information, patient-specific disease mechanisms can be uncovered, enabling the selection of tailored therapies that target the underlying molecular factors.

Scalable analytical platforms such as mass spectrometry and next-generation sequencing play a critical role in the success of multi-omics integration. These technologies enable the generation of high-throughput data that is essential for clinical feasibility. Advanced bioinformatics tools that can process and visualize complex data sets are equally important. These tools enable clinicians to go from raw data to clinically meaningful results such as risk stratification models or treatment recommendations.

Translational research forms the bridge between multi-omics discoveries and their clinical application [87]. Pilot studies are crucial to assess the feasibility of omics-guided interventions. For example, clinical trials examining lipidomic profiles in HF patients could assess their ability to predict therapeutic response to drugs such as sodium-glucose co-transporter 2 inhibitors. Such studies provide proof of concept and pave the way for large-scale implementation. In addition, registries that incorporate omics data, such as the Heart ‘omics’ in AGEing (HOMAGE) project, offer valuable resources for validating biomarkers and exploring their impact on clinical outcomes [88].

Education and training are essential components of this integration. Clinicians need to be equipped with the skills to effectively interpret omics data. This can be achieved through targeted workshops, certification programs and the integration of omics modules into medical education. This not only prepares current physicians for the evolving landscape of HF management, but also fosters a culture of innovation among future healthcare providers.

From a regulatory perspective, the introduction of multi-omics technologies must be aligned with strict guidelines to ensure patient safety and data integrity. Regulators need to establish a clear framework for the approval of omics-based diagnostics and therapies, considering the unique challenges posed by the complexity and novelty of these approaches. Ethical considerations, particularly around data protection and informed consent, are also critical. Patients must be fully informed of the implications of their participation in omics studies and robust data protection measures must be in place to protect sensitive information.

The integration of biomarkers and multi-omics into HF management offers significant opportunities to reduce healthcare costs. Early diagnosis enabled by cfDNA and miRNA panels can prevent costly hospitalizations by allowing timely interventions. By stratifying patients based on molecular profiles, clinicians can better target therapies, reducing the trial-and-error approach that often characterizes HF treatment. For example, lipidomic analysis could identify patients who are more likely to respond to certain drug classes, avoiding unnecessary spending on less effective options.

In addition, multi-omics data can optimize resource allocation within healthcare systems. Hospitals can prioritize intensive care for patients with high-risk omics profiles, ensuring that resources are allocated to those most likely to benefit [89]. This is consistent with value-based care models that emphasize cost efficiency and improved quality-adjusted life years (QALYs) [90]. In addition, as omics technologies advance, the cost of high-throughput platforms is expected to decrease, further increasing their cost-effectiveness.

**Fig. 3:**
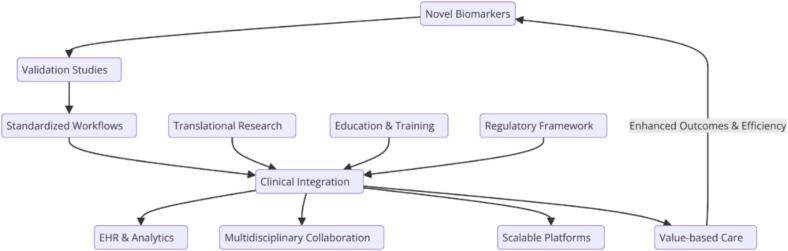
Outlines the pathway for integrating novel biomarkers into clinical practice for HF management. It begins with the validation of biomarkers like cfDNA, miRNAs, ST2, and Galectin-3 through rigorous studies and the establishment of standardized workflows. Translational research bridges discovery and application, while education and regulatory frameworks support their adoption. Central to this is clinical integration, enabled by multidisciplinary collaboration, EHR analytics, and scalable platforms. Aligned with value-based care, this process aims to improve diagnostic precision, personalize therapies, and enhance patient outcomes, transforming HF management.

Personalized medicine, which relies on multi-omics, represents a paradigm shift in HF care. It offers the potential not only for better clinical outcomes, but also for a more sustainable healthcare model. By addressing the molecular heterogeneity of HF, omics-guided approaches enable targeted interventions that maximize therapeutic efficacy while minimizing unnecessary costs. As these technologies are increasingly integrated into clinical practice, they promise to redefine the standard of HF care and ensure that patients receive the most precise and effective treatment (Fig. 3).

## 5 Future directions

The treatment of HF is changing, driven by advances in multi-omics technology and the discovery of new biomarkers. While these developments are very promising, there are still some critical gaps and challenges that need to be addressed to enable effective translation into clinical practice. Key areas we need to focus on include large-scale validation, technological innovation and equitable accessibility.

Large-scale validation studies and international collaborations are essential to determine the clinical utility of biomarkers such as cfDNA, miRNAs, ST2 and Gal-3. The heterogeneity of HF requires validation in diverse populations representing different ethnicities, age groups and HF subtypes, such as HFrEF and HFpEF. Longitudinal studies with longer follow-up periods can provide insights into the predictive power of biomarkers, their role in therapeutic decisions and their correlation with clinical outcomes, including mortality and hospitalization. Collaborative initiatives such as the Heart ‘omics’ in AGEing (HOMAGE) project exemplify how pooling resources and data can accelerate progress. Expanding such efforts to underrepresented regions would further enhance our understanding of HF in various healthcare settings.

Advances in point-of-care (POC) technologies are critical to the integration of multi-omics into routine HF management. Recent innovations such as the UC-LFS platform with dual-color up conversion nanoparticles enable simultaneous measurement of multiple biomarkers, including BNP and ST2, facilitating early diagnosis and real-time monitoring. The integration of POC devices with smartphone applications offers additional benefits by enabling remote monitoring and rapid treatment adjustments, which is particularly beneficial in the treatment of chronic HF. To ensure widespread adoption, future research should focus on developing cost-effective and scalable tools that are accessible in resource-limited settings.

Policy changes are needed to ensure accessibility and equity in the use of multi-omics technologies. Governments and health organizations need to invest in infrastructure to support omics-based diagnostics, especially in low- and middle-income countries. Policy makers should include omics diagnostics in insurance coverage to reduce the financial burden and promote uptake. Ethical and regulatory frameworks are also critical to address concerns about privacy, security and the responsible use of omics data. Outreach programs and subsidies may be needed to ensure that advances benefit marginalized and underserved populations.

The convergence of multi-omics with artificial intelligence (AI) and machine learning (ML) offers transformative opportunities for HF care. AI-powered algorithms can uncover patterns in omics data that may be missed by traditional methods, enabling the discovery of new biomarkers and therapeutic targets. By integrating multi-omics data with clinical and lifestyle information, AI can support the development of precision medicine models that are tailored to the individual patient. Cloud-based platforms for omics data analysis can further democratize access to advanced computing resources and enable healthcare providers in resource-constrained environments to leverage cutting-edge technologies.

Finally, interdisciplinary collaboration is critical to realizing the full potential of multi-omics for HF management. Synergy between researchers and clinicians can accelerate the translation of omics findings into actionable insights, while public-private partnerships can drive innovation and reduce costs. Involving patients in the development and implementation of omics technologies can also improve adoption, particularly through educational initiatives that emphasize their benefits for HF care.

## 6 Conclusion

HF is a global health problem with high prevalence, morbidity and mortality, underlining the need for innovative approaches. Emerging biomarkers such as cfDNA, miRNAs, ST2 and galectin-3 promise earlier diagnosis, better risk stratification and non-invasive monitoring, marking a shift towards precision medicine. Multi-omics technologies, including lipidomics, metabolomics, proteomics and genomics, enable a multidimensional understanding of HF and thus the discovery of new biomarkers, therapeutic targets and personalized treatment strategies. However, challenges such as the complexity of data integration, high costs and ethical concerns regarding equity and privacy remain. Collaboration between researchers, clinicians and policy makers is critical to validate biomarkers, build infrastructure and ensure equitable access. The integration of advanced biomarkers and multi-omics offers transformative potential for HF management, paving the way for personalized, predictive cardiovascular care and significantly improving patient outcomes.

## CRediT authorship contribution statement

**Jose Mesquita Bastos:** Writing – review & editing, Investigation, Formal analysis. **Beatriz Colaço:** Writing – review & editing, Investigation, Formal analysis. **Rui Batista:** Writing – review & editing, Investigation. **Cristina Gavina:** Writing – review & editing, Investigation. **Rui Vitorino:** Writing – original draft, Methodology, Investigation, Formal analysis, Data curation, Conceptualization.

## Funding information

This work was supported by the Portuguese 10.13039/501100001871Foundation for Science and Technology (FCT), 10.13039/501100000780European Union, QREN, 10.13039/501100008530FEDER, and 10.13039/501100011929COMPETE for funding Institute of Biomedicine (iBiMED) (UIDB/04501/2020, POCI-01-0145-FEDER-007628), Cardiovascular R&D Centre (UnIC, UIDP/00051/2020).

## Declaration of competing interest

The author declare he has no conflict of interest.

## Data Availability

None.
